# Perceived Crises and Preparedness Gaps in Operating Room Nursing: A Qualitative Study of Training Priorities With Nurses and Educators

**DOI:** 10.1155/jonm/5539605

**Published:** 2026-06-04

**Authors:** Inas D. Redjem, Arnaud Huaulme, Pierre Jannin, Estelle Michinov

**Affiliations:** ^1^ Univ Rennes 2, LP3C (Laboratoire de Psychologie: Cognition, Comportement, Communication)-EA 1285, Rennes, F35000, France; ^2^ Univ Rennes, INSERM, LTSI (Laboratoire du Traitement du Signal et de L’Image)-UMR 1099, Rennes, F35000, France, inserm.fr

**Keywords:** crisis management, focus group, nurse anaesthetists, operating room, perceived preparedness, perioperative nurses

## Abstract

**Background:**

The operating room (OR) is a complex, high‐stress environment in which crisis situations place considerable demands on staff and compromise patient care. Despite the availability of crisis management training, existing research has predominantly relied on objective measures of effectiveness rather than examining nurses′ perceived preparedness to manage crises.

**Aim:**

This study aims to explore nurses’ perceptions of crisis management in the OR, specifically what they perceive as a crisis situation, and which crises they consider important but feel insufficiently prepared to manage. Examining these dimensions allows for the identification of crisis situations representing priority areas for OR nurses’ training.

**Methods:**

This exploratory qualitative study used focus group interviews with certified nurse anaesthetists, perioperative nurses, and nurse educators from anaesthesia and perioperative nursing programmes. Seven focus groups were analysed using qualitative content analysis, using an inductive approach with limited quantification.

**Results:**

Five categories of crisis situations were identified: (1) interpersonal dynamics; (2) staffing and equipment constraints; (3) errors or incidents during the care process; (4) unexpected external events; and (5) deterioration in the patient’s condition. Participants reported feeling least prepared to manage interpersonal conflicts, emotional demands, and unexpected external events.

**Conclusion:**

This study identifies two critical training gaps. First, while nurses receive substantial training in procedural and technical crisis management, they perceive a significant lack of nontechnical skills training, particularly regarding interpersonal dynamics. Second, training related to low‐frequency, high‐impact crises appears insufficient.

## 1. Introduction

The operating room (OR) is a complex and high‐stress environment in which surgical teams are confronted with unexpected or rapidly evolving crisis situations that place considerable demands on staff and require continuous, coordinated actions among multiple healthcare professionals [1]. In recent crisis management literature, a crisis is defined as ‘a process of weakening or degeneration that can culminate in a disruptive event affecting the actor’s (i.e., individual, organisation, and/or community) normal functioning’ ([2], p. 735). Two main conceptualisations of crises coexist: crisis as an event and crisis as a process [2]. From the perspective of crisis as a disruptive event (e.g., [3]), a crisis is characterised by surprise and unpredictability and resembles an exceptional situation for which no pre‐established response exists. Within the process‐orientated view (e.g., [4, 5]), a crisis unfolds over time through accumulating conditions that eventually trigger a disruptive event. However, in both conceptualisations, such situations place professionals in scenarios for which they have received little or no prior training. They must therefore improvise appropriate responses within a complex, dynamic, and time‐pressured environment to ensure patient safety. When crises are poorly managed, the consequences can be severe: Adverse events (AEs) occurring during the perioperative period are among the leading causes of preventable disability and mortality [6]. Thus, effective crisis management is a central component of safe surgical practice.

These findings have accelerated the development of structured training initiatives targeting crisis management in the OR. Over the past several years, international recommendations and training programmes have been developed to enhance quality of care and reduce the incidence of AEs in the OR [7]. A recent systematic review also highlighted the increasing availability of crisis management training modules specifically designed for OR settings [8]. However, the availability of training does not guarantee perceived preparedness in real‐world practice, and this distinction remains underexplored. In this context, perceived preparedness refers to an individual’s subjective sense of their capacity to respond effectively to crisis situations, encompassing both perceived competence across different dimensions of crisis response [9] and psychological preparedness, which is defined as the ability to ‘manage responses to maintain cognitive function and behavioural performance, and to reduce risk’ ([10], p. 2).

Despite the centrality of this construct to crisis management, there has been little recent research on nurses′ perceptions of crisis in the OR, and no studies have specifically examined their perceived preparedness for crisis management in this context. Existing research on perioperative crisis situations has largely focused on AEs defined as unexpected incidents resulting from medical management, including equipment or communication failures and procedural mistakes [11]. Additionally, one qualitative study on incidents in the OR showed that incidents reported by nurse anaesthetists include poor communication and teamwork within the OR and issues stemming from noncompliance with established guidelines and protocols [12]. Overall, the broader literature on OR crises has predominantly relied on objective measures rather than individuals′ subjective sense of their capacity to respond to such situations. How OR nurses themselves define crisis situations, and how they perceive their preparedness to respond across these situations, remains unexplored.

To address this gap, this qualitative study explores how nurses perceive and experience crisis situations in the OR. More specifically, this study seeks to

1. explore what is perceived as a crisis situation by nurses in the OR
2. examine which crises nurses consider important but feel insufficiently prepared to manage

Taken together, these investigations allow for the identification of crisis situations representing priority areas for OR nurse training.

## 2. Methods

This study employed an exploratory descriptive qualitative design using focus group interviews [13, 14], which allow for the exploration of participants’ beliefs and experiences [15] and are more efficient and cost‐effective than individual interviews [16]. Analysis was conducted within an inductive, interpretive framework, with limited quantification to support the qualitative findings. This study is reported in accordance with the Consolidated Criteria for Reporting Qualitative Research (COREQ; [17]; see Supporting Information 1).

### 2.1. Ethical Considerations

This study received approval from the university’s ethics committee (approval number #2024‐041). Participants provided written informed consent and were informed that participation was strictly voluntary, with no incentives, no involvement of direct supervisors in recruitment or data collection, and no professional consequences for withdrawal. They were also informed that the moderator was a PhD candidate in psychology conducting research on simulation‐based training for nontechnical skills (NTS) and that the aim of the study was to explore perceived training needs for crisis management in the OR.

### 2.2. Recruitment

Participants were recruited using snowball sampling through email dissemination by medical and paramedical professionals across three French hospitals, including both public and private institutions. The inclusion criteria for this focus group study were (1) the ability to read and write in French and (2) at least 1 year of prior work experience in the OR as a nurse anaesthetist or in another perioperative nursing role.

This study included nurse anaesthetists, perioperative nurses, perioperative nursing students, and nurse educators from anaesthesia and perioperative nursing programmes. The term perioperative nurse is used in this study to refer to participants trained or working as scrub nurses, circulating nurses, or surgical assistants, regardless of their specific role during surgery. Recruiting nurse educators alongside practising nurses and perioperative nursing students allows for the collection of complementary perspectives on crisis management competencies. Practising nurses contribute up‐to‐date, experience‐based insights into OR practice and crisis situations, while educators offer a broader pedagogical view on training needs and curriculum coverage. The perspectives of perioperative nursing students capture perceptions at an early stage of professional socialisation, thereby complementing the accounts of more experienced practitioners and educators.

### 2.3. Data Collection

The focus groups were conducted between October 2023 and May 2024, either at participants’ workplaces or at the research laboratory. Data collection continued until thematic sufficiency was reached; that is, until no new themes emerged and additional data only further illustrated previously identified categories [18].

Each focus group was homogeneous in professional composition and included three to six participants. In particular, students were not grouped with educators or practising clinicians to reduce potential power dynamics that could inhibit free expression. However, due to logistical constraints, one focus group included both nurse anaesthetists and perioperative nurses. All focus groups were facilitated by the first author, who is trained in qualitative methods and used a standardised semistructured interview guide (Supporting Information 2). The guide was developed based on a review of the relevant literature and was initially piloted with perioperative nursing students. Data collected during this piloting phase were not included in the analysis. A silent observer was present during each focus group to take supporting notes that helped identify points not fully addressed during the discussion and to be raised at the end of the session. Observer notes were not coded analytically but supported the transcription by clarifying instances of overlapping speech.

**FIGURE 1:**
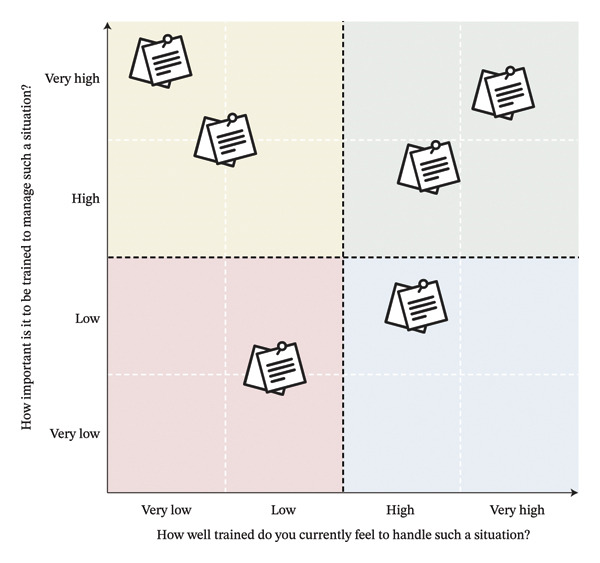
Two‐axis prioritisation diagram.

Each focus group began with a presentation of the study aim, followed by the signing of an informed consent form and the completion of a brief sociodemographic questionnaire. Participants then individually reflected for 10–15 min and documented crisis situations they had encountered in the OR. These situations were collectively discussed, using a series of open‐ended questions exploring (1) whether participants had encountered the situation; (2) emotional responses during the crisis; (3) perceived preparedness at the time; (4) resolution strategies; (5) professional roles in the resolution process; and (6) perceived importance of training for each situation. Finally, a prioritisation exercise was conducted using a two‐axis diagram to identify training priorities (see Figure 1). Each focus group collectively positioned reported situations on the diagram according to two criteria: (1) perceived importance of training for the situation and (2) perceived preparedness to manage it. Positioning was determined by group consensus. Each session concluded with a synthesis of the discussion and time for final questions.

### 2.4. Data Analysis

All focus groups were audio‐recorded and lasted approximately 1 h. Recordings were automatically transcribed using the open‐source speech recognition model WHISPER [19], and then reviewed for accuracy by the first author and a research assistant.

Qualitative content analysis [20] was conducted using an inductive approach, with categories and subcategories derived directly from the data. Limited quantification was used to examine the frequency and distribution of categories across focus groups.

Data analysis proceeded iteratively to establish thematic sufficiency. Focus groups were analysed progressively, with preliminary categories refined after each round of coding. The first author read all transcripts to establish familiarity with the data. The first and last authors, both trained in qualitative methods, and then inductively developed preliminary categories and subcategories. These categories were discussed with two perioperative nursing students to ensure accurate interpretation of OR‐specific terminology and context. After the fifth focus group, no new thematic categories emerged. The sixth and seventh focus groups confirmed thematic sufficiency by providing only further illustrations of the main categories without generating new themes.

The prioritisation diagram was analysed systematically (see Figure 1). Each diagram was divided into quadrants representing high and low levels of perceived importance and perceived preparedness using horizontal and vertical midlines. These axes were further subdivided into five‐point scales ranging from very low to very high. Prioritisations from each focus group were first analysed separately and then aggregated into a composite diagram. This allowed identification of thematic clusters and comparison across professional profiles. Crisis situations were categorised according to their position relative to both axes, yielding combined categories of perceived importance and preparedness.

### 2.5. Research Team and Reflexivity

Data familiarisation, coding, and content analysis were conducted by the first and last authors. The first author, who also served as moderator of all focus groups, is a female doctoral candidate in psychology, specialising in NTS in OR settings, with 3 years of collaborative research experience with surgical teams and nursing education programmes. The last author is a full professor of psychology specialising in social and organisational psychology, with extensive experience working with nurses and OR teams. She supervised the analytical process and contributed expertise in human factors, interpersonal dynamics, and team functioning. The second and third authors are researchers in computer and data science with longstanding collaborations in OR research and contributed to analytic triangulation by challenging disciplinary assumptions and supporting methodological coherence. The authors had no hierarchical or clinical relationship with participants. A small proportion of nursing students had previously encountered the first author in educational settings but had no ongoing relationship at the time of data collection. In addition to the listed authors, the broader project team included perioperative nurses, nurse anaesthetists, and anaesthesiologists. The transcripts and analyses were not returned to participants for formal member checking due to their clinical or pedagogical commitments. However, preliminary and final findings were presented to this extended team during research meetings, as well as educator stakeholders, allowing informal validation and feedback. No substantive corrections or disagreements with the interpretations were reported.

## 3. Results

### 3.1. Characteristics of Participants

In total, seven focus groups were conducted, comprising 28 participants. Seven participants were certified nurse anaesthetists; 11 were perioperative nurses (either certified or students with OR experience); four were nurse educators from anaesthesia nursing programmes; and six were nurse educators from other OR nursing programmes.

Participants had a median age of 45.5 years (range = 27–64), and 75% were female. The median OR experience was 11.5 years (range = 1–42). All participants had at least 1 year of OR experience, including students. In the French context, students enrolled in perioperative nursing programmes may work in OR settings and perform perioperative nursing functions prior to formal certification. Accordingly, all students in the sample had substantial and active exposure to the OR environment. Table 1 provides details of participants’ demographic characteristics.

**TABLE 1:** Characteristics of participants in each focus group.

Focus group	Current status	Gender	Age	OR experience	Time away from the OR	Workplace setting	NTS training
1	Nurse anaesthetist educators	Male	57	4	19	Public hospital	No
		Female	42	7	0		Yes
		Male	63	12	19		Yes

2	Nurse anaesthetist	Male	37	8	0	Private clinic	No
	Perioperative nurse	Female	51	28	0		Yes
	Perioperative nurse	Female	54	33	0		Yes
	Nurse anaesthetist	Female	34	10	0		Yes
	Nurse anaesthetist	Female	42	6	0		No

3	Perioperative nurse educators	Female	64	42	12	Public hospital	Yes
		Female	57	35	5	Private clinic	Yes
		Male	46	21	4	Public hospital	No

4	Perioperative nursing students	Female	32	9	0	Private clinic	Yes
		Female	27	2	0	Public hospital	Yes
		Female	29	7	0	Private clinic	Yes

5	Nurse anaesthetists	Female	47	13	0	Public hospital	Yes
		Female	43	8	0		No
		Male	50	16	0		No
		Female	47	16	0		Yes

6	Perioperative nurses	Female	46	20	0	Public hospital	Yes
		Female	35	11	0		No
		Female	41	1	0		No
		Female	43	5	0		No
		Female	39	14	0		No
		Female	43	11	0		Yes

7	Nurse anaesthetist educator	Male	48	25	0	Public hospital	Yes
	Perioperative nurse educator	Female	53	25	5		Yes
	Nurse anaesthetist educator	Female	54	6	16		No
	Nurse anaesthetist educator	Male	45	21	0		Yes

*Note:* The NTS training column indicates participants who received prior training in nontechnical skills.

### 3.2. Crisis Situations in the OR

To explore what nurses perceive as a crisis situation in the OR, a total of 124 crises reported across the seven focus groups were analysed. Content analysis identified five main categories (see Table 2): (1) interpersonal dynamics; (2) errors during patient care; (3) patient’s condition; (4) staffing and equipment issues; and (5) unexpected external events. These categories were reported across all nursing profiles, suggesting a shared experience of crisis in the OR, regardless of specialisation or level of experience.

**TABLE 2:** Summary of content analysis categories and subcategories of OR crisis situations.

Main categories	Subcategories	Number of situations	Focus group coverage	Reporting profiles	Illustrative quotes
Interpersonal dynamics	Conflicts	*n* = 19	6/7	Nurse anaesthetist educators, nurse anaesthetists, perioperative nurse educators, perioperative nurses	‘You’re afraid he’s going to hit you, like… You can feel that there’s physical tension. It’s not going well, because either you feel like he doesn’t like us, or, actually, we just don’t get along with him.’ (FG 6)
	Emotion regulation	*n* = 17	6/7	Nurse anaesthetist educators, nurse anaesthetists, perioperative nurses, perioperative nursing students	‘She started crying because she just couldn’t do it anymore. She kept crying, saying, “I’m not going to manage [the procedure].”’ (FG 7)
	Communication	*n* = 9	6/7	Nurse anaesthetist educators, nurse anaesthetists, perioperative nurse educators, perioperative nurses	‘Give me this, give me that, do this’ she throws everything on the floor, that’s it.’ (FG 2)

Errors during patient care	Errors in decision‐making	*n* = 6	4/7	Perioperative nurse educators, perioperative nursing students, nurse anaesthetists, nurse anaesthetist educators	‘The surgeon says, “Anyway, take your time, it’s not a high‐stakes procedure” […] But then I see the operation going on and on and on, and the longer it went on, the more it put the patient at risk.’ (FG 7)
	Pharmacovigilance	*n* = 4	3/7	Perioperative nursing students, nurse anaesthetists, nurse anaesthetist educators	‘The Curare was mistakenly injected instead of Hypnovel before spinal anaesthesia. The patient became unresponsive. It was a mistake.’ (FG 4)
	Protocol violations	*n* = 7	4/7	Nurse anaesthetist educators, nurse anaesthetists, perioperative nurses	‘The children were brought to the recovery room […] that’s where the confusion happened […] the wrong name had been labelled on a bed. So they assumed, “This is child so‐and‐so,” since that’s the name on the bed. But in reality, it wasn’t the right child.’ (FG 1)

Staffing and equipment	Equipment‐related problems	*n* = 16	5/7	Nurse anaesthetists, perioperative nurse educators, perioperative nursing students, perioperative nurses, nurse anaesthetist educators	‘During the procedure, at one point, a crucial element was missing […] since it was a loaned instrument, a one‐time loan, there was no spare, no second set available.’ (FG 3)
	Organisational breakdowns	*n* = 10	5/7	Perioperative nurse educators, perioperative nursing students, perioperative nurses, nurse anaesthetist educators	‘There’s a procedure currently underway… and a code red being called in the room next door… I’m on my own, I have to handle both.’ (FG 4)
	Lack of training	*n* = 9	5/7	Perioperative nurse educators, perioperative nursing students, perioperative nurses, nurse anaesthetist educators	‘Lack of knowledge about the procedure from a colleague… It’s a bit like having someone incompetent in the situation.’ (FG 3)

Unexpected external events	Infrastructure failures	*n* = 4	3/7	Perioperative nurses, nurse anaesthetists, nurse anaesthetist educators	‘I ended up extubating a patient using a flashlight and monitoring her clinical status for half an hour.’ (FG 2)
	Security threats	*n* = 3	2/7	Perioperative nurse educators, nurse anaesthetists	‘In the emergency OR, we heard that a suspicious bag had been left… not inside the operating room itself, but out in a courtyard behind it. And basically, the room where I was working was just 10 m away from the bag.’ (FG 5)
	Environmental disruptions	*n* = 3	2/7	Perioperative nurses, nurse anaesthetist educators	‘I had a fire case one time’ (FG 7)
	Public health emergencies	*n* = 2	2/7	Perioperative nurses, nurse anaesthetist educators	‘It was… the COVID crisis. At first, we had two ICU beds set up in the OR during the first wave. Then, during the second wave, when all surgeries were cancelled again, just like the first time, the entire OR staff was reassigned to the intensive care unit.’ (FG 6)

Patient’s condition		*n* = 15	6/7	Nurse anaesthetist educators, nurse anaesthetists, perioperative nurse educators, perioperative nurses, perioperative nursing students	‘It was the gas embolism we had a month ago.’ (FG 2)

*Note:* The number of situations column indicates the number of situations (out of the 124 total situations reported) coded within each subcategory. The focus group coverage column indicates the number of focus groups in which at least one situation related to the subcategory was mentioned.

#### 3.2.1. Interpersonal Dynamics

Interpersonal dynamics constituted the most frequently reported crisis category, with situations distributed comparably between nurse anaesthetists and perioperative nurses. These situations encompassed conflicts, emotion management, and communication failures. Conflicts most commonly arose between physicians and nurses, highlighting interprofessional tension, while conflicts between surgeons and perioperative nurses were attributed to the physical and procedural proximity within the sterile field. Emotion‐regulation crises involved intense affective states such as anger, anxiety, fear, and panic, as well as leadership breakdowns (e.g., inaction, hesitation, or an inability of the individual with formal authority within the team to respond effectively). They were predominantly attributed to physicians, most of the time the surgeon. Finally, a set of situations reported involved communication breakdowns ranging from incomplete, ambiguous, or uncoordinated exchanges between professionals with potentially serious clinical consequences to contextual or logistical constraints such as poor acoustic conditions in the OR leading to misunderstandings, repeated requests, and execution errors.

#### 3.2.2. Staffing and Equipment Issues

Staffing‐ and equipment‐related issues were the second most reported crisis category, predominantly by perioperative nurses. Equipment‐related problems were the most common subcategory and included missing, unavailable or malfunctioning devices. Organisational breakdowns, including staffing shortages and unclear task distribution, were also reported, as well as lack of training or competence among colleagues. Across these subcategories, participants emphasised that resource constraints, whether human or material, can create tension and undermine teamwork.

#### 3.2.3. Errors During Patient Care

Crises attributed to human error were another type of crisis identified, most frequently by nurse anaesthetists. They included protocol violations, such as errors in patient identification, wrong‐site surgery or anaesthesia, and incorrect surgical counts; pharmacovigilance failures such as incorrect medication administration; and cognitive errors in decision‐making related to tunnel vision by surgeons.

#### 3.2.4. Unexpected External Events

Participants described some situations involving external and unanticipated events disrupting OR activity and triggering crisis situations. These crises included infrastructure failures, security threats, environmental disruptions, and public health emergencies reminiscent of COVID‐19. They were described as destabilising due to the need to adapt rapidly outside standard protocols, underscoring the importance of flexibility, preparedness, and collective decision‐making in high‐stakes environments. Though less frequent, these situations were reported to a similar extent by nurse anaesthetists and perioperative nurses.

#### 3.2.5. Patient’s Condition

Crises associated with the patient’s condition involved sudden deterioration or unexpected surgical complications (e.g., haemorrhage or cardiac arrest) and were frequently reported across focus groups and in similar proportions across nursing profiles.

### 3.3. Perceived Preparedness and Training Priorities

For each category of crisis reported by participants, perceived preparedness and importance were examined. Overall, findings consistently revealed a relationship between the technical nature of a crisis and participants’ sense of preparedness: OR nurses felt most prepared for clinically acute and procedurally defined situations and least prepared for those involving interpersonal or emotional demands and unexpected external events. Supporting information 3 provides a detailed comparative table of perceived importance and preparedness for each professional profile recruited.

Interpersonal dynamics represented the category with the widest gap between perceived importance and preparedness. Across all professional groups, conflict situations were consistently perceived as high to very high in importance, while perceived preparedness varied markedly between groups. Among practising nurses, perceived preparedness ranged from high to very low, while educators consistently reported low to very low preparedness. Emotion regulation showed a similar pattern: universally regarded as high to very high in importance while perceived preparedness varied. Practising perioperative nurses demonstrated the widest range of preparedness, spanning from very high to low, while nurse anaesthetists reported slightly more moderate levels of preparedness, and students reported low preparedness. Communication‐related situations showed comparatively higher and more consistent preparedness, though nurse anaesthetist educators reported low preparedness despite very high importance ratings. This category thus emerges as the clearest training priority across all profiles.

Errors during patient care were consistently perceived as high to very high in importance but revealed role‐specific preparedness profiles. Nurse anaesthetists reported high to very high importance and high to very high preparedness for pharmacovigilance, consistent with its centrality in anaesthesia practice. Protocol violations, perceived as highly important across all groups, showed inverted preparedness patterns compared with pharmacovigilance. Perioperative nurses reported very high preparedness, while nurse anaesthetists reported only moderate to high preparedness. However, errors in decision‐making, especially those attributable to surgeons, generated a sense of unpreparedness across profiles, as participants perceived these situations as exceeding their professional authority, and high to very high preparedness was reported when errors occurred within the scope of the nurses’ practice.

Staffing and equipment issues showed divergent preparedness patterns. Equipment‐related problems were consistently perceived as highly important, reflecting a shared understanding of their direct impact on patient safety and procedural continuity, but perceived preparedness was generally higher among perioperative nurses, educators, and students compared to nurse anaesthetists. Organisational breakdowns were generally perceived as less important and associated with low preparedness across most groups, with participants often describing a sense of limited agency in the face of systemic constraints.

Unexpected external events represented the other category, with interpersonal dynamics, for which the gap between importance and preparedness was most pronounced across all groups, showing moderate to very low preparedness reported despite very high perceived stakes. This category therefore represents the second critical training gap.

Finally, patients’ condition crises were consistently perceived as very high in importance across all groups. However, perceived preparedness showed variability across professional profiles, with anaesthesia nurses feeling more prepared than nurses working, teaching or studying in perioperative roles that felt moderately prepared, consistent with the emphasis placed on acute clinical management in anaesthesia training.

## 4. Discussion

**FIGURE 2:**
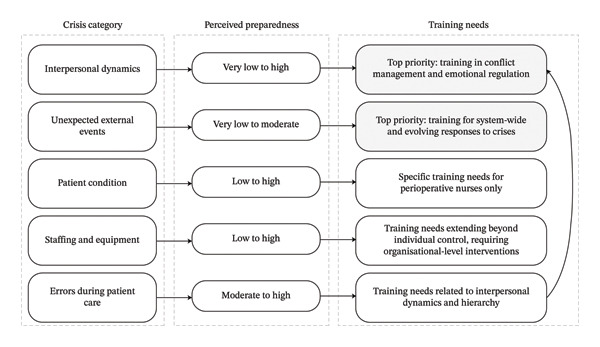
Synthesis of crisis categories, perceived preparedness, and identified training priorities among OR nurses.

This qualitative study aimed to explore perceptions of crisis management in the OR by addressing two questions: (1) what is perceived as a crisis situation by nurses in the OR? and (2) what crises do OR nurses consider important but feel insufficiently prepared to manage? Results revealed five categories of situations perceived as crises by OR nurses: interpersonal dynamics, errors during patient care, patient’s condition, staffing and equipment issues, and unexpected external events. Additionally, OR nurses expressed feeling less prepared for NTS‐related crises and unexpected external events, positioning these areas as clear priorities for OR nurse training (see Figure 2).

The five categories identified as perceived crises in the OR are consistent with previous research that has sought to identify what is characterised as intraoperative AEs or incidents [11, 12]. Altogether, they reflect the multifaceted nature of perioperative crisis and align with the distinction between crisis as an event and crisis as a process [2]. Some situations (e.g., interpersonal conflicts, equipment malfunctions, and unexpected external events) were described as abrupt, unpredictable, and poorly anticipated events, requiring improvisation under time pressure and in socially complex contexts. Others (e.g., organisational dysfunction, staffing shortages, or perceived lack of competence among colleagues) reflect progressive degradation embedded in routine practice. This distinction highlights the need for educational models emphasising both rapid response to disruptive events and anticipation within complex healthcare systems. These findings also offer a nurse‐generated OR crisis typology based on lived experience, extending beyond incident‐based classifications to include subjectively experienced crises.

A second aim of this study was to identify the types of crises nurses consider important but feel insufficiently prepared to manage. Here, interpersonal dynamics emerged as a major training priority across nursing profiles, with NTS consistently identified as critical to crisis preparedness [21–23]. Within this domain, conflict management represented the most critical issue in perioperative settings, confirming previous findings highlighting communication and teamwork failures in the OR [12, 24] and disruptive behaviours in perioperative contexts [25]. Nurses identified conflicts between professions as particularly challenging, which appeared to reflect hierarchical dynamics within the OR team, confirming earlier work describing the OR as a highly stratified environment in which role legitimacy and speaking‐up remain constrained [24, 26]. Emotion regulation under pressure emerged as a related but distinct critical gap, substantially influencing team dynamics. It involved leadership breakdowns and intense emotional responses, aligning with previous research documenting the emotional landscape of OR professionals [27, 28].

Taken together, these findings suggest that conflict management and emotion regulation skills develop unevenly through individual experience and personal coping strategies rather than through formalised training. Educators reported lacking pedagogical tools to prepare learners for conflict management, while students struggled to identify conflict as a crisis or were reluctant to discuss such experiences. Additionally, the absence of educator reports and students’ low preparedness regarding emotion regulation suggests a significant training gap in which emotional demands are not yet supported by structured educational frameworks.

From a nursing management perspective, this gap is critical. Reliance on informal learning may perpetuate uneven competency development and expose teams to avoidable risk by normalising and under‐discussing interpersonal tensions [29], even though effective teamwork in healthcare requires deliberate speaking‐up in contexts where hierarchical norms are strong and psychological safety may be limited [30]. Accordingly, explicit and profession‐sensitive training programmes should target interpersonal skills, with particular emphasis on conflict management and emotional regulation [31, 32]. Concrete components may include scenario‐based speaking‐up simulations, structured debriefings focused on hierarchical communication, emotion‐regulation training modules, and in situ interprofessional crisis drills within the OR environment. Simulation‐based and interprofessional learning approaches appear particularly relevant for strengthening speaking‐up behaviours, collective coordination, and leadership under pressure [24, 26, 33, 34].

Unexpected external events (e.g., pandemic‐like situations) constituted the second key training priority, particularly for rare but high‐impact situations requiring system‐wide, prolonged, and evolving responses [4]. Similarly, organisational breakdowns were often conceptualised as structural issues beyond individual control, which may contribute to reduced perceived agency and lower preparedness. These findings suggest that the more systemic a crisis, the lower the perceived preparedness, regardless of perceived importance. Preparedness appeared highest when crises intersected directly with established professional competencies and clear role boundaries and lowest when crises required cross‐sectoral coordination, rapid organisational adaptation, or responses extending beyond the OR. Overall, these results highlight the need to expand crisis preparedness beyond clinical and interpersonal domains to include system‐level resilience, emergency coordination, and large‐scale disruption management.

Overall, these findings highlight that crisis preparedness in perioperative settings extends beyond clinical competence to encompass interpersonal dynamics, organisational structures, and systemic constraints. For nursing managers, improving crisis preparedness requires both individual‐level training and organisational interventions aimed at strengthening teamwork climate and coordination capacity. At the educational level, the systematic gap between educators’ perceived importance and low preparedness suggests that training the trainers may be a critical leverage point. Without adequate pedagogical tools, educators may remain unable to translate recognised training priorities into effective learning experiences. Future training models should therefore benefit from integrating structured NTS curricula, simulation‐based interprofessional learning, and explicit strategies addressing hierarchy, professional legitimacy, and speaking‐up behaviours within OR teams.

### 4.1. Limitations and Future Research

This study has some limitations from which future research directions emerge. First, the sample size was relatively small, with most participants employed in a large academic hospital. It was also not possible to stratify practising professionals according to clinical experience, limiting transferability of the results. Recruiting larger and more diverse samples stratified by clinical experience and institutional setting could clarify how organisational context and professional hierarchies shape crisis perception and preparedness. Nevertheless, this study provides in‐depth insights into how OR nurses conceptualise crisis situations and identify perceived gaps in preparedness. Second, recruitment was conducted using snowball sampling via email dissemination by medical and paramedical professionals, which may have introduced self‐selection bias. Participants who volunteered may have had a particular interest in crisis management. However, the data revealed substantial variability in perceived importance and preparedness across professional groups, suggesting that participation was not restricted to a single highly motivated or engaged profile. Third, the composition of the focus groups may have influenced participants’ discourse. The authors’ academic positioning as external to clinical practice facilitated critical examination of implicit practices and hierarchical norms but also required clarification of technical terminology during discussion, which may have directed attention towards interpersonal and organisational dimensions of crisis. In addition, some focus groups included educators. While their perspectives enriched the discussion by providing a training‐orientated perspective, their contributions may have relied more heavily on retrospective reflections rather than recent clinical practice.

Beyond addressing these methodological limitations, future intervention research is needed for NTS‐related crises trainings. Future research should evaluate the impact of conflict management, emotion regulation, and speaking‐up training programmes using validated measures of teamwork climate and safety climate (e.g., Safety Communication, Operational Reliability, and Engagement Questionnaire [35]), as well as behavioural observation tools adapted to perioperative crisis scenarios (e.g., Hospital Survey on Patient Safety Culture [36]). Combining these measures during simulated crisis exercises would allow more comprehensive assessment of training effectiveness.

### 4.2. Conclusion

This qualitative study provides a detailed description of crisis situations encountered by nurses working in the OR and highlights critical gaps in crisis preparedness training. Although participants generally felt confident in managing crises aligned with procedural and technical competencies, they consistently reported low levels of preparedness for situations involving interpersonal conflict, emotional regulation, and hierarchical tension, highlighting the central role of human factors in surgical patient safety. Preparedness for low‐frequency but high‐impact external events also emerged as a priority. These findings support the need to broaden existing models of crisis preparedness by integrating structured NTS curricula, including conflict management and emotion regulation, as well as dedicated simulation‐based training.

## Funding

This work was supported by state aid managed by the French National Research Agency under the France 2030 programme, bearing the reference ANR‐21‐DMES‐0001.

## Conflicts of Interest

The authors declare no conflicts of interest.

## Supporting Information

Additional supporting information can be found online in the Supporting Information section.

## Supporting information


**Supporting Information 1** Supporting Information 1: COnsolidated criteria for REporting Qualitative research (COREQ) checklist, indicating for each item the corresponding page reference in the main manuscript.

**Supporting Information 2** Supporting Information 2: Semistructured interview guide employed in this study, including exemplar prompts designed to elicit participants’ perceptions and experiences.

**Supporting Information 3** Supporting Information 3: Table of perceived preparedness and importance for each category and subcategory of crisis.

## Data Availability

The data that support the findings of this study are available upon request.
