# Supplementary material for: Perceived Crises and Preparedness Gaps in Operating Room Nursing: A Qualitative Study of Training Priorities With Nurses and Educators
Source: J Nurs Manag. 2026 Jun 4;2026:5539605. doi: 10.1155/jonm/5539605 (PMC13238243; doi:10.1155/jonm/5539605)
Supplement: Supplementary file 2 — Supporting Information 2 Supporting Information 2: Semistructured interview guide employed in this study, including exemplar prompts designed to elicit participants’ perceptions and experiences. [file JONM-2026-5539605-s003.docx]

## **Focus Group Interview Guide**

**Date and location :
Moderators:
Observer:**

| **WELCOME AND INTRODUCTION** | 5 minutes |
| --- | --- |
| - Welcome and thank you - Distribution of informed consent forms - Information about audio recording - Completion of demographic questionnaire | |

| **OPENING REMARKS** | 2 minutes |
| --- | --- |
| The session will last approximately one hour. The objective is to identify training needs for crisis management in the operating room.  Discussion ground rules:   - Allow everyone to speak and listen actively - Avoid debate or confrontation - No judgment and no criticism | |

| **AGENDA OVERVIEW** | 5 minutes |
| --- | --- |
| 1. Each participant individually notes crisis situations they have experienced (on sticky notes) 2. Group discussion of the reported situations 3. Prioritisation activity based on the group’s input | |

| **STEP 1 – Reporting Crisis Situations** | 10 minutes |
| --- | --- |
| Instructions: *“What are your experiences with crisis situations in the operating room? Take a moment to recall several examples, and write each one on a separate sticky note.”*  Participants write on sticky notes, which are then displayed on a board. | |

| **STEP 2 – Group Discussion** | 25 minutes |
| --- | --- |
| Review EACH sticky note with the WHOLE group.  Discussion questions:   - Have you experienced this type of situation? - What did you feel during the situation? - How prepared did you feel to manage it? - How was the crisis resolved? - What role did each professional play in the resolution? (e.g. Who took the lead?) - Do you think nurses are adequately prepared to face this type of crisis? | |

| **STEP 3 – Prioritisation Activity** | 10 minutes |
| --- | --- |
| Instructions: *“We will now move to a prioritisation task. You may now discuss and share your views, please do so respectfully and without interrupting others. How well do you currently feel trained for each of these situations? How important do you feel it is to be able to manage such situations ? And if you were to receive training for crisis situations in the OR, how would you rank the listed situations in order of priority?”* | |

| **WRAP-UP AND CLOSING** |  |
| --- | --- |
| - Final questions - Summary of key themes discussed | |
