# Supplementary material for: Perceived Crises and Preparedness Gaps in Operating Room Nursing: A Qualitative Study of Training Priorities With Nurses and Educators
Source: J Nurs Manag. 2026 Jun 4;2026:5539605. doi: 10.1155/jonm/5539605 (PMC13238243; doi:10.1155/jonm/5539605)
Supplement: Supplementary file 3 — Supporting Information 3 Supporting Information 3: Table of perceived preparedness and importance for each category and subcategory of crisis. [file JONM-2026-5539605-s001.docx]

**Supplementary Material 1**

*Perceived Preparedness and Importance for Each Category and Subcategory of Crisis*

| **Main categories** | **Subcategories** | **Perceived preparedness** | **Perceived importance** | **Illustrative quotes** |
| --- | --- | --- | --- | --- |
| Interpersonal dynamics | Conflicts | Nurse anaesthetists: low to high | Nurse anaesthetists: high to very high | *“Trained or not... we’ll try to handle it […] otherwise it’s going to go badly.”* (FG 5) |
|  |  | Nurse anaesthetist educators: low to very low | Nurse anaesthetist educators: very high | *“In personality and in, um, I was going to say the ability to position oneself and put oneself forward, that’s where we have the fewest levers.”* (FG 1) |
|  |  | Perioperative nurses: very low to high | Perioperative nurses: high to very high | *“It’s very important, yes. It can create real problems not to know how to manage a crisis in an operating room. But no, we are not trained.”, “Our training makes us take a position. That is also part of our responsibilities. But we are really trained.”* (FG 6) |
|  |  | Perioperative nurse educators: very low to low | Perioperative nurse educators: high to very high | *“I think we never really feel fully trained, and it's very important.”* (FG 3) |
|  |  | Perioperative nursing students: none | Perioperative nursing students: none | */* |
|  | Emotion regulation | Nurse anaesthetists: low to moderate | Nurse anaesthetists: high to very high | *“It concerns everyone”* (FG 5) |
|  |  | Nurse anaesthetist educators: very low to low | Nurse anaesthetist educators: very high | *“I think it’s extremely important and that we are very poorly trained.”* (FG 7) |
|  |  | Perioperative nurses: low to very high | Perioperative nurses: very high | “*Knowing how to de-escalate a conflict. I think that, in itself, that’s it. Yes.”, “As for me, I had communication training because I worked in the emergency department, and we had training on that, which told us that in front of you, you have someone who raises their voice and gets angry.”* (FG 6) |
|  |  | Perioperative nurse educators: none | Perioperative nurse educators: none | / |
|  |  | Perioperative nursing students: very low to moderate | Perioperative nursing students: high to very high | *“We don’t have any theoretical classes that teach us how to manage our emotions or how to respond in those situations.”* (FG 4) |
|  | Communication | Nurse anaesthetists: moderate | Nurse anaesthetists: moderate | *“It’s important, but not vital.”* (FG 2) |
|  |  | Nurse anaesthetist educators: low | Nurse anaesthetist educators: very high | *“That’s the key. It’s the school that will teach you how to position yourself, I think […] It’s that I knew how to position myself because I knew why I was doing things. So, in fact, I knew how to stand up to it.”* (FG 1) |
|  |  | Perioperative nurses: high | Perioperative nurses: very high | *“A feeling of training in that area” [high].* (FG 6) |
|  |  | Perioperative nurse educators: moderate | Perioperative nurse educators: moderate | *“It is less discussed [in the training], actually. It’s what reaction to have.”* (FG 3) |
|  |  | Perioperative nursing students: none | Perioperative nursing students: none | */* |
| Errors during patient care | Errors in decision-making | Nurse anaesthetists: high to very high | Nurse anaesthetists: high to very high | *“It’s important.”, “Do we feel very trained… [thinks] Yeah… rather yes!”* (FG 6) |
|  |  | Nurse anaesthetist educators: very low | Nurse anaesthetist educators: very high | *“And are we ready for that? Not at all. We are not trained at all.”* (FG 7) |
|  |  | Perioperative nurses: none | Perioperative nurses: none | / |
|  |  | Perioperative nurse educators: very high | Perioperative nurse educators: very high | *“We’re trained to be… we’re kind of a lookout on the field, a complementary observer. For me, that’s the ultimate level of what a nurse should be.”* (FG 3) |
|  |  | Perioperative nursing students: moderate | Perioperative nursing students: very high | *“It also depends on the degree of error.”* (FG 4) |
|  | Pharmacovigilance | Nurse anaesthetists: very high | Nurse anaesthetists: very high | *“It’s a rare and very serious situation, and we’ve all been trained for it.”* (FG 5) |
|  |  | Nurse anaesthetist educators: moderate to high | Nurse anaesthetist educators: high to very high | *“It’s very important. Because it can, in fact, jeopardize a professional’s career later on. But we normally train on risks. Yes, very well.”* (FG 7) |
|  |  | Perioperative nurses: none | Perioperative nurses: none | / |
|  |  | Perioperative nurse educators: none | Perioperative nurse educators: none | / |
|  |  | Perioperative nursing students: low | Perioperative nursing students: high | *“it’s a life-threatening risk, it’s going to be as important as the life-threatening risk, but it’s on the anaesthetists’ side, but actually, uh…”* (FG 4) |
|  | Protocol violations | Nurse anaesthetists: high | Nurse anaesthetists: high | *“That is extremely important… When there are consequences, but if we make a mistake… Well, for me, for me we are trained with the checklist.”* (FG 2) |
|  |  | Nurse anaesthetist educators: moderate | Nurse anaesthetist educators: high | *“Well in any case, they have, they have the procedural tools, that’s clear, that’s clear. They have the procedural tools, that’s for sure”.* (FG 1) |
|  |  | Perioperative nurses: very high | Perioperative nurses: very high | *“Our training makes us take a position. We are really trained.”* (FG 6) |
|  |  | Perioperative nurse educators: none | Perioperative nurse educators: none | / |
|  |  | Perioperative nursing students: none | Perioperative nursing students: none | / |
| Staffing and equipment | Equipment-related problems | Nurse anaesthetists: low | Nurse anaesthetists: high | *“We know the alternatives.”* (FG 2) |
|  |  | Nurse anaesthetist educators: very high | Nurse anaesthetist educators: very high | *“Well trained, but very important. It’s only equipment. We are surrounded by techniques.”* (FG 7) |
|  |  | Perioperative nurses: high | Perioperative nurses: high | *“That’s important! But it’s what we were saying, we know how to find a solution together. With the knowledge we have. What can replace one thing with another. Well, that, that’s experience.”* (FG 6) |
|  |  | Perioperative nurse educators: high | Perioperative nurse educators: high | *“We are trained essentially to prevent it.”* (FG 3) |
|  |  | Perioperative nursing students: high | Perioperative nursing students: high | *“Well I would say that it’s still quite important. Well I find that we are still quite well trained in risk prevention and and in dealing with lack of equipment, so uh…”* (FG 4) |
|  |  |  |  |  |
|  | Organisational breakdowns | Nurse anaesthetists: none | Nurse anaesthetists: none | */* |
|  |  | Nurse anaesthetist educators: very low | Nurse anaesthetist educators: very low | *“I managed. But afterwards, even if we take responsibilities, I think we are trained, still. We are going to find solutions, we are going to call.”* (FG 7) |
|  |  | Perioperative nurses: low | Perioperative nurses: high | *“It puts us in an awkward position as professionals.”* (FG 6) |
|  |  | Perioperative nurse educators: low | Perioperative nurse educators: moderate | *“That is more of a management dynamic. Yes, yes, management.”* (FG 3) |
|  |  | Perioperative nursing students: low | Perioperative nursing students: moderate | *“That’s something the executives handle.”* (FG 4) |
|  | Lack of training | Nurse anaesthetists: none | Nurse anaesthetists: none | */* |
|  |  | Nurse anaesthetist educators: moderate | Nurse anaesthetist educators: moderate | *“We can manage. I would say, still, for me, it’s the somewhat emergency situations, where, precisely, we are trained for degraded situations where we are somewhat trained.”, “In surgery, we can’t compensate. We see that it’s happening, and if we can discreetly ask for someone to stop by the OR to help out, fine. Otherwise, we know it’s going to go south.”* (FG 7) |
|  |  | Perioperative nurses: moderate | Perioperative nurses: low | *“It was important and there was no training. Important to know how to manage it, but you can’t know how to manage it when you’ve just arrived.”* (FG 6) |
|  |  | Perioperative nurse educators: high | Perioperative nurse educators: high | *“We are going to end up managing it anyway on a daily basis.”* (FG 3) |
|  |  | Perioperative nursing students: low to moderate | Perioperative nursing students: low to high | “*Even if we can be sure, or know where we want to go, uh… I think that sometimes it makes us doubt and we maybe won’t dare, actually, uh… Well, say something.”* (FG 4) |
| Unexpected external events | Infrastructure failures | Nurse anaesthetists: high | Nurse anaesthetists: high | *“It’s extremely important! Yes yes, we are trained regarding being connected, which socket to plug the ventilator into, an oxygen cylinder… it’s the safety elements, actually.”* (FG 2) |
|  |  | Nurse anaesthetist educators: high | Nurse anaesthetist educators: low | *“They know how to handle it.”* (FG 7) |
|  |  | Perioperative nurses: low | Perioperative nurses: high | *“Yes, it’s vital, not on the IBODE side, huh [the training].”* (FG 2), *“We don’t have control over it. We don’t have any other solution except stopping the scheduled programme.”* (FG 6) |
|  |  | Perioperative nurse educators: none | Perioperative nurse educators: none | */* |
|  |  | Perioperative nursing students: none | Perioperative nursing students: none | */* |
|  |  |  |  |  |
|  | Security threats | Nurse anaesthetists: moderate | Nurse anaesthetists: high | *“Yeah, it’s important if it’s a real… No, and then even that has consequences, it has real consequences anyway.”* (FG 5) |
|  |  | Nurse anaesthetist educators: none | Nurse anaesthetist educators: none | / |
|  |  | Perioperative nurses: none | Perioperative nurses: none | / |
|  |  | Perioperative nurse educators: moderate | Perioperative nurse educators: high | *“There, we are not trained and I think the problem is not so much for us.”* (FG 3) |
|  |  | Perioperative nursing students: none | Perioperative nursing students: none | / |
|  | Environmental disruptions | Nurse anaesthetists: none | Nurse anaesthetists: none | / |
|  |  | Nurse anaesthetist educators: very low to low | Nurse anaesthetist educators: very low to low | *“We are less trained, maybe.”* (FG 7) |
|  |  | Perioperative nurses: moderate | Perioperative nurses: very high | *“Ah well, that, we are not trained at all. I mean, we are not trained in situ. That’s often what I criticize. We have training but outside the operating room. It’s extremely important, still.”* (FG 6) |
|  |  | Perioperative nurse educators: none | Perioperative nurse educators: none |  |
|  |  | Perioperative nursing students: none | Perioperative nursing students: none |  |
|  | Public health emergencies | Nurse anaesthetists: none | Nurse anaesthetists: none | / |
|  |  | Nurse anaesthetist educators: very low | Nurse anaesthetist educators: very high | *“The worst crisis situation that has ever existed, which shook everyone, which required us to reorganize everything. Are we ready? Are we trained? I don’t think so.”* (FG 7) |
|  |  | Perioperative nurses: very low | Perioperative nurses: very high | *“Not trained at all, and yet it’s super important [to manage the COVID-19 crisis].”* (FG 6) |
|  |  | Perioperative nurse educators: none | Perioperative nurse educators: none | */* |
|  |  | Perioperative nursing students: none | Perioperative nursing students: none | */* |
| Patient’s condition |  | Nurse anaesthetists: very high | Nurse anaesthetists: very high | *“For me, we are trained and… it’s important.”* (FG 5) |
|  |  | Nurse anaesthetist educators: very high | Nurse anaesthetist educators: very high | *“For me, it’s even the most important.”* (FG 7) |
|  |  | Perioperative nurses: moderate | Perioperative nurses: very high | *“It’s the maximum level of importance.”* (FG 6) “*But since it’s something we don’t do every day, for example, we are less trained, less competent than the nurse anaesthetist who can do it every day.”* (FG 6) |
|  |  | Perioperative nurse educators: low | Perioperative nurse educators: very high | *“I think there are situations for which we are never really prepared.”* (FG 3) |
|  |  | Perioperative nursing students: moderate | Perioperative nursing students: very high | *“It’s as important as it gets.”* (FG 6) |
